# Annotations

**Published:** 1890-12-06

**Authors:** 


### Annotations.

**The Sunday Fund.**

The annual report of the Hospital Sunday Fund has just been issued. The grand total of all the collections taken on its behalf is what its friends wish first to be satisfied about. The report will give them considerable satisfaction on this head. The total amount collected in 1890 was £42,814 16s. 9d., a sum which exceeded by £1,070 3s. 10d. the largest amount ever collected in any previous year, and was more by £16,000 than the amount taken in 1879. But the real increase of the total actually collected at the churches and chapels is larger than appears here, and amounts, in fact, to between three and four thousand pounds. There has been a considerable falling off in the amount accruing from legacies during the past year. If it had not been for this accidental failure, the Fund would have reached about £45,000. Legacies are always regarded by hospital managers as the most fluctuating of all their sources of income. Last year having been decidedly barren in this respect, we may hope that in the present year there will be a large and abundant crop. We cannot forbear calling the attention of the supporters of the Fund to the economy of the management. The total expenditure in the collection and distribution of this large sum of money was £1,544 2s. 8d., or a little over three and a-half per cent. of the gross receipts. It is evident, therefore, that the Hospital Sunday collection presents to benefactors the most economical of all the methods of giving to hospitals. It is clear from the report that the Fund is steadily growing, as it well deserves to grow. The number of contributing places of worship last year was considerably larger than on any previous occasion. The council distributed close upon £40,000 to 113 hospitals and 56 dispensaries, and they set apart a little over £2,000 for the purchase of surgical appliances. Of the 169 institutions thus aided, sixteen, or about one in ten, were required by the committee to offer explanations on matters of an apparently unsatisfactory character. These were dealt with, and three out of the sixteen were recommended for reduced awards. One hospital received no grant at all from the council. Four hospitals thought themselves entitled to larger awards than formerly, and three of them successfully maintained their contention before the Council. The Council recognised the value of the services to the Fund of Mr. Jonathan Hutchinson, F.R.S., and tendered to that gentleman a cordial vote of thanks. Mr. Hutchinson, who was then President of the Royal College of Surgeons, inaugurated the annual proceedings by an address at the Mansion House, which was as practically useful as it was wide-ranging and philosophical. The Council also passed an equally grateful vote of thanks to the representatives of the press for their intelligent and discriminating advocacy of the Fund. There is no doubt that Hospital Sunday has become a highly popular institution, and that both the press, the public, and hospital managers desire for it a wider range and greater efficiency. We would suggest that next year's collections should reach as a minimum £50,000.

**The Steady Briton.**

Stubborn, obstinate, common-sense—that is the diagnostic mark of the typical Briton. The Frenchman shrugs his shoulders, and smiles airily at our immovable stolidity; the German wonders through his learned spectacles for a moment, and then plunges into the profoundest recesses of medical, metaphysical, and omni-scientific haystacks, bogs, and mile-deep sea-bottoms. There he loses himself and is happy, until he re-appears, after some longer or shorter interval, to enlighten the world by means of doubtful grammar, inextricable logic, and profound speculations, which, like the ancient Melchizedek, are "without beginning of days or end of life." The Briton, meanwhile, stands squarely on his two feet, keeps his eyes open, and works or waits as the occasion seems to demand. This somewhat long introduction is apropos of a visit made by Dr. Saundby, of Birmingham, to Dr. Koch at Berlin, and of a similar visit made independently by Dr. Conan Doyle, of Southsea. Dr. Saundby reports that in his judgment Dr. Koch has been forced by the press to divulge his discovery before it was really ripe for scientific discussion. Dr. Conan Doyle is of opinion that Dr. Koch has not discovered a cure for consumption. Now Dr. Saundby is well known to the medical profession, both by his clinical work and his contributions to medical literature. He is a solid, judicious, and honest physician; and his words are of undoubted weight. As we said in our last issue, we are anxious to avoid taking either one or the other side in the momentous controversy which Dr. Koch has raised. But it is plain that sides are being taken. The Bacteriologists, who may be called speculative medical scientists, are singling themselves out, or ranging themselves on the side of Dr. Koch. The Clinicians, or practical physicians who engage in the hand-to-hand fight with disease and death at the bed-side, seem more and more inclined, as time goes on, to doubt, to question, and to deny. The best medical minds in Great Britain remain steady. They have not been in the least moved by the deluge of popular excitement. All the British medical papers last week uttered practically the same note. We will wait, and we will see, they said in effect; and when we see, we will believe. Medicine is not like metaphysics: its principles and its advances are not to be settled in the region of logic: they are to be settled objectively, and before the eyes; and so plainly and unmistakably settled that no man can say no, because the facts say yes. Great Britain at the present time has every reason to be proud of her doctors. They are in the van of all medical progress, and eager to move forward; but they will not plunge aside into any region of doubt and uncertainty where danger lies for their patients. Forward they will go, but only where there is solid foothold for the feet, and light by which they can guide their way. This we hold to be in the highest degree scientific and rational, and, from the standpoint of the patient, the very attitude of mind to be desired.

**Paupers and Their Food.**

The Worcester Guardians have let it be known to the world what they think of the paupers over whom they bear rule. According to the *Globe* of November 22nd, the guardians some little time ago were desirous of laying in a stock of flour for the paupers' use; and accordingly they invited tenders for "best thirds." One particular contractor however, sent in a sample of what was called "eighth grade," which is said to be the lowest quality of flour put upon the market. The guardians, it is to be presumed, saw the sample and approved it, with the result that a stock of "eighth grade" was sent in. On being examined, however, the flour was found to be absolutely unfit for use, even for paupers' use; although it was not stated to be in anyway "inferior to sample." There must be paupers, and apparently there must be guardians. It does not require any very elevated social rank or a high degree of mental power to make a guardian. But it does require some sort of conscience and sense. Even a guardian should know that human beings are almost on as high a level as pigs and horses; and no guardian of any brains would buy the very lowest quality of food stuffs which the market could produce for his pigs and horses; because he would know that it would not pay to do so. It is not possible to imagine any grown-up man, guardian or otherwise, who has not heard the common "saw" of the market that "the best things are always the cheapest." But whether the best things are always the cheapest or not, it is quite certain that the worst are always the dearest. It will be observed that we are not asking the Worcester Guardians to show a little kindly feeling towards their human brothers and sisters whose lives are compelled to be spent in the dull and dreary monotony of the workhouse. We do not ask for such feeling from those guardians, because gentle feeling is the offspring of intelligence; and it is certain that intelligence cannot exist in the minds of persons who purchase flour which is only fit to be thrown away. Self-interest is the faculty to be appealed to: it is a faculty which no living creature fails to be endowed with. Pigs, crocodiles, sharks, and all human beings, lunatics and idiots included, are invested with the faculty of self-interest; and, therefore, we are sure that the Worcester Guardians are not without it. Our advice to those gentlemen is, that the next time they contemplate the starving of their paupers, they shall do it intelligently, so that if it accomplishes no other end, it shall at least save the ratepayers' pockets. There is nothing so stupid as bungling. Even if men want to do things that are altogether vulgar, and mean, and detestable, they should still try to put the seasoning of a little brains into them.

